# The Frequency of Poor Sleep Quality in Patients With Diabetes Mellitus and Its Association With Glycemic Control

**DOI:** 10.7759/cureus.11608

**Published:** 2020-11-21

**Authors:** Rabia Farooque, Fivzia Herekar, Sundus Iftikhar, Muhammad Junaid Patel

**Affiliations:** 1 Internal Medicine, Liaquat University of Medical and Health Sciences, Jamshoro, PAK; 2 Internal Medicine and Infectious Diseases, The Indus Hospital, Karachi, PAK; 3 Maternal and Child Health Program, Interactive Research and Development, Karachi, PAK; 4 Internal Medicine, The Indus Hospital, Karachi, PAK

**Keywords:** pittsburgh sleep quality index (psqi), diabetes mellitus, glycemic control, cardiometabolic risk

## Abstract

Background and objective

Emerging evidence suggests that sleep problems are more common among individuals with diabetes mellitus (DM) than in the general population; these sleep issues are associated with poor glycemic control and they negatively affect the overall prognosis of the disease by increasing cardiometabolic risk. Our study aimed to determine the frequency of poor sleep quality and its association with glycemic control among Pakistani adult patients with DM.

Methods

This prospective cross-sectional study was conducted at the outpatient department (OPD) of The Indus Hospital (TIH), Karachi, and included 329 participants. To be eligible, participants had to be 14 years or older, should have been visiting the OPD at TIH for six months or more to seek treatment for DM, and had to give informed consent. Participants were assessed for poor sleep quality using the Pittsburgh Sleep Quality Index (PSQI) score and glycemic control using HbA1C levels ascertained through electronic health record review, with higher HbA1C levels reflecting poorer glycemic control.

Results

Two-thirds of the participants were females (n=212; 64.4%), and approximately 90% of the participants were married (n=292; 88.8%); 57% (n=188) of the participants were found to have poor sleep quality (PSQI of >5) and 233 (70.82%) had poor glycemic control (HbA1C of >7). Interestingly, no significant difference was observed in the PSQI scores between participants with controlled diabetes and those with uncontrolled diabetes.

Conclusion

Based on our findings, there is a high prevalence of sleep disturbance among Pakistani adults with DM, and we believe this necessitates the fostering of sleep-promoting interventional research in the country, as it might be highly rewarding and would positively affect the overall prognosis for diabetes by improving cardiometabolic risks. However, our results did not indicate any significant association between sleep quality and glycemic control. Further research should be conducted to explore the association between sleep disturbance and DM in Pakistani adults, by employing objective measures of sleep quality and involving a larger sample of individuals with DM to determine if these results hold true.

## Introduction

Sleep is a natural and essential phenomenon for humans, but sleep problems are common and are likely to increase due to multi-pronged issues, mostly due to stress stemming from the rapid modernization of life or health concerns, etc. The constant and increasing demand for more shift work and similar activities has contributed immensely to sleep problems among humans [1]. Research suggests that sleep is a modifiable behavior and at least seven hours of sleep per day is essential for optimum physical health and overall wellbeing in adults [2,3]. Scholarly research in sleep has revealed that short sleep duration is associated with poor immunity, increased inflammatory biomarkers, and increased incidence of obesity, hypertension (HTN), diabetes mellitus (DM), cardiovascular diseases (CVD), and stroke [1,4-7]. There is growing evidence to suggest that decreased sleep duration and/or quality adversely affect glucose regulation and increase the risk of developing DM, and this risk is comparable to that of other, traditional risk factors related to DM [8-13]. Research also suggests that sleep plays a major role in glycemic control, with decreased sleep duration and/or quality adversely affecting glycemic control among individuals with DM [14-18]. Sleep problems are significantly more common among individuals with DM than in the general population, which may be due to the disease itself or its complications [19]. Fortunately, research has also demonstrated that sleep habits can be improved, and obtaining adequate sleep is associated with beneficial effects on insulin sensitivity and energy metabolism [20,21]. The assessment of sleep quality is, therefore, of paramount importance among individuals with DM as management of sleep problems can be highly rewarding [19]. However, there is a paucity of consistent, high-quality data about sleep in low- and middle-income countries (LMIC); the available data show that about one-third of the adult population in LMIC report disturbed sleep and/or poor sleep quality, which is comparable to the situation in high-income countries [22].

In Pakistan, DM prevalence is alarmingly high and is estimated to be at 26.3%. Pakistan is currently placed at number four in the ranking of nations with the highest prevalence of DM and is estimated to reach number three by 2030 [23,24]. Research determining the association between sleep and glycemic control among racially/ethnically diverse populations is scarce, and no study has examined this relationship in Pakistani adults with DM so far. In light of this, we aimed to determine the frequency of poor sleep quality and its association with glycemic control in Pakistani adults with DM.

## Materials and methods

This prospective cross-sectional study was conducted at The Indus Hospital (TIH), Karachi, a free-of-cost tertiary care facility. A priori sample size of 329 was calculated using the OpenEpi software (www.OpenEpi.com) with the following assumptions: 69% prevalence of poor sleep quality in adults with DM [25]; the desired precision of 5%. The institutional review board of The Indus Hospital approved all study protocols. The inclusion criteria were as follows: patients aged 14 years of age or older of either gender, who had been visiting the OPD at TIH for six months or more to seek treatment for DM. The exclusion criteria were as follows: patients diagnosed with DM within the past year; those who were not able to answer the sleep questionnaire due to psychiatric illness; those who had not checked their HbA1c within the past three months of the interview; patients with known hemoglobinopathies or anemia, chronic kidney disease/end-stage renal disease; and patients who were pregnant. All eligible participants gave informed consent either on their own or through their guardians if they were below the age of 18 years. The study involved interviews with the participants that lasted 20-30 minutes, along with an analysis of their electronic health record data. The interviews assessed patients' demographics, medical history regarding comorbidities, duration of their DM, and medication use.

The interview included a validated Urdu translation of the Pittsburgh Sleep Quality Index (PSQI) [26,27] to assess sleep quality. It comprised 19 questions categorized into seven components, each graded on a scale ranging from 0 to 3, generating a global sleep quality score ranging from 0 to 21. A global PSQI score of >5 distinguished poor sleepers from good sleepers. Glycemic control was assessed via HbA1C levels obtained from participants’ electronic health records. Medication use was divided into diabetes-specific and medication for other health issues; diabetes-specific medications were further classified into insulin and oral antidiabetic drugs. Body mass index (BMI) was calculated as kg/m^2^, based on patients' weight and height recorded during their outpatient department (OPD) visits. All data were collected using a pre-designed questionnaire.

## Results

A total of 329 participants were enrolled in the study. Of them, two-thirds were females (n=212; 64.4%) and approximately 90% were married (n=292; 88.8%). More than half of the participants were Urdu-speaking (181; 55%), and 308 (93.6%) of the participants were Muslims The mean age was 52.13 years [standard deviation (SD)=10.876], and the mean BMI was 28.7 kg/m^2^ (SD=7.46). Three-fourth (n=232; 70.5%) of the participants had at least some school education, while 97 (29.5%) were uneducated. Only 99 (30.1%) of the participants were employed, out of which 54 (16.4%) were employed full time; 35 (10.6%) were self-employed, and six (1.8%) were employed part-time. Of the participants who were not doing any kind of job, the majority were female homemakers (n=182; 55.3%); 22 (6.7%) were retired, and nine (2.7%) were either bed-bound or dependent on others for their daily activities.

On average, participants had been diagnosed with diabetes for eight years (mean=7.95 years; SD=6.97). The mean HbA1c was 8.13% (SD=1.8; range=4.8-16.7%). With regard to DM medication, more than half were on oral antidiabetic medication (189; 57.4%); 101 (30.7%) were taking both oral medication and insulin, and 39 (11.9%) were only on insulin, and there were no significant differences in their PSQI scores. Among those who were on oral medication, all (n=290; 100%) were taking metformin, and 197 (67.9%) were also using sulfonylurea.

Three-fourth of the participants (76.9%) had one or more comorbidities; HTN (n=203; 80.2%) and ischemic heart disease (n=73; 28.9%) were the most commonly observed comorbid conditions. Nearly two-thirds (61%; n=201) of the participants reported the use of aspirin; half (49.2%; n=162) of the participants reported the use of angiotensin-converting enzyme inhibitors/angiotensin II receptor blockers (ACEI/ARBs), 40.4% (n=133) were using statins, and 19.4% (n=64) were using beta-blockers. Twenty-three (7%) participants admitted to using medicines without prescriptions. Among those, 13 (56.5%) reported the use of homeopathic medicines, followed by allopathic medicine use by eight (34.8%) and herbal medicine use by six (26.1%).

The mean PSQI score of the study participants was 6.6 (SD=3.2), and 57% (n=188) had a global PSQI score of ≥5, indicating poor quality of sleep; however, in response to the specific question ‘During the past month, how would you rate your sleep quality overall?’, two-thirds (n=217; 66%) of the participants rated their overall sleep quality as good, and 37 (11.2%) rated it as very good. Average sleep latency was 35.4 minutes (SD=34.3), and the mean duration of sleep was 6.5 hours (SD=1.6). Almost half (50.46%; n=166) of the participants were not getting the minimum recommended duration of sleep (<7 hours) (Table 1).

**Table 1:** Demographic and health-related characteristics of participants – overall and stratified by sex SD: standard deviation; BMI: body mass index; HbA1c: glycated hemoglobin; PSQI: Pittsburgh Sleep Quality Index

Variables	Full sample (n=329), mean (SD)	Male (n=117), mean (SD)	Female (n=212), mean (SD)
Age in years	52.13 (10.88)	55.12 (10.65)	50.47 (10.66)
Years of schooling	6.74 (5.17)	8.89 (4.88)	5.45 (4.91)
BMI, kg/m^2^	28.7 (7.46)	27.1 (5.5)	29.7 (8.2)
Duration of diabetes mellitus, years	7.95 (6.97)	7.48 (6.43)	8.21 (7.25)
HbA1c, %	8.13 (1.78)	8 (1.7)	8.2 (1.8)
PSQI score	6.6 (3.2)	6 (2.9)	6.9 (3.3)
Sleep hours/night	6.5 (1.6)	6.6 (1.5)	6.4 (1.7)

Interestingly, no significant difference was seen in PSQI scores between the participants with controlled diabetes and those with uncontrolled diabetes. In addition, no significant difference was observed in PSQI scores based on gender, marital status, occupation, and BMI, as shown in Table 2.

**Table 2:** Difference in PSQI score according to participants' characteristics *Kruskal-Wallis test PSQI: Pittsburgh Sleep Quality Index; IQR: interquartile range; HbA1c: glycated hemoglobin; BMI: body mass index

Distribution of PSQI scores				
	N	Median (IQR)	Min-max	P-value*
Marital status				
Single	7	7 (3-14)	2-15	0.181
Married	292	6 (4-8)	1-17	
Widowed	27	7 (6-9)	3-6	
Divorced	1	6 (6-6)	6-6	
Occupation				
Employed full-time	54	5.5 (3.8-8)	2-16	0.581
Employed part-time	6	5 (4-6)	4-6	
Unemployed and currently looking for work	3	6 (6-0)	6-7	
Unemployed and currently not looking for work	13	6 (5-9.5)	2-14	
Retired	22	5 (4-7.3)	2-14	
Homemaker	182	6 (4-9)	2-17	
Self-employed	35	6 (4-10)	1-15	
Unable to work	9	5 (4.5-9.5)	1-16	
HbA1c				
Glycemic control	149	6 (4-8)	1-16	0.801
Uncontrolled diabetes	177	6 (4-8)	1-17	
BMI				
Under-weight	8	6 (4.3-7)	4-11	0.688
Normal	45	5 (4-8)	2-15	
Over-weight	40	5 (5-6.8)	1-14	
Pre-obese	112	6 (4-9)	2-17	
Obese	124	6 (4-8)	1-16	

## Discussion

To our knowledge, this is the first study examining sleep quality among Pakistani adults with DM. There is a paucity of research regarding sleep in Pakistan. Our study revealed that 57% (n=188) of Pakistani adults with DM suffered from poor sleep quality (PSQI of >5), and 50.46% (n=166) were not getting the recommended duration of sleep (<7 hours), suggesting that the majority was suffering from sleep disturbance. These findings demand a need for sleep-promoting interventional research among Pakistani adults with DM, as it might be highly rewarding. Short sleep duration is associated with poor immunity, increased inflammatory biomarkers, and increased incidence of obesity, HTN, DM, CVDs, and stroke, thereby contributing to significant morbidity and mortality and negatively impacting the quality of life and posing an increased financial burden to both individuals and society [4-7,11].

There is growing evidence to suggest that decreased sleep duration and/or quality adversely affect glycemic control in adults with DM [14,16-18], even though a few studies have demonstrated no significant association between sleep duration and/or quality and glycemic control in adults with type II DM [25,28]. Our study found no significant differences in sleep quality between participants with controlled diabetes and those with uncontrolled diabetes. However, Knutson et al.'s [14] study on African American adults and Ohkuma et al.'s [17] study on Japanese adults with type 2 DM demonstrated a significant association between participants’ self-reported sleep measures and glycemic control. Knutson et al. [29], in another study, found a clinically significant association between sleep disturbance (defined by sleep fragmentation) and insulin resistance. In this study, sleep measures were assessed objectively via actigraphy. Full et al.'s [28] study found no significant association between sleep duration and glycemic control in Hispanic adults with uncontrolled type 2 DM. Similarly, Rajendran et al.'s [25] study in South India found no correlation between sleep quality and glycemic control. Further research is needed to explore this sleep-glycemic control relationship in Pakistan, by using a larger sample size and objective measures of sleep, as sleep is a modifiable lifestyle behavior and we are facing a diabetes epidemic. Also, since sleep curtailment is a growing problem and likely a contributor to the diabetes epidemic, improving sleep quality may improve the overall prognosis for diabetes by reducing cardiometabolic risk.

## Conclusions

Our study revealed a high prevalence of sleep disturbance among Pakistani adults with DM, which demands a need for sleep-promoting interventional research, as it might be highly rewarding, possibly affecting the overall prognosis for diabetes by improving cardiometabolic risks. However, we could not find any significant association between sleep quality and glycemic control. Further research is needed to explore the association between sleep disturbance and DM in Pakistani adults, by using objective measures of sleep quality and a larger sample of individuals with DM to determine if our findings hold true.
